# Comparing the predictive performance of different lymph node staging systems for postoperative overall survival in patients with ampullary carcinoma

**DOI:** 10.3389/fsurg.2023.1002411

**Published:** 2023-02-27

**Authors:** Xun Li, Lin Chen, Junli Li

**Affiliations:** ^1^ Department of Blood Transfusion, Jingzhou Hospital Affiliated to Yangtze University, Jingzhou, China; ^2^ Department of Gastroenterology, Jingzhou Hospital Affiliated to Yangtze University, Jingzhou, China

**Keywords:** ampullary adenocarcinoma, SEER, overall survival, lymph node stage systems, predictive performance

## Abstract

**Aim:**

This study was to analyze and compare the predictive performance of the 7th and the 8th edition American Joint Committee on Cancer (AJCC) N staging system, lymph nodes ratio (LNR) and log odds of positive lymph node (LODDS) for the survival of patients with ampullary carcinomas (ACs).

**Method:**

This retrospective cohort study included patients with primary ACs after surgery from the Surveillance, Epidemiology, and End Results (SEER) 2004–2015. Univariate and multivariate Cox proportional hazard models were used. The study population was divided into a training set and a testing set in a ratio of 7–3. The C-index and area under the curve (AUC) were used to compare the predictive performance of the four staging on overall survival (OS) in the training set and the testing set.

**Results:**

A total of 7,480 patients with primary ACs (1,178 survived and 1,128 dead) were in this study. The average follow-up time was 41.1 months. N1 stage and N2 stage of the 8th edition AJCC N staging system, LNR staging (0–0.3), LNR (>0.3), LODDS (−2.4 to −0.8) and LODDS (>−0.8) were associated with OS in AC patients after adjusting for age, race, pT stage, tumor size, grade, radiation, and insurance. The C-index of the 7th AJCC N staging was significantly lower than the C-index of the 8th AJCC N staging in the training set [0.608 vs. 0.629, *P* < 0.001] and testing set [0.635 vs. 0.658, *P* < 0.001]. The C-index of the LODDS staging was significantly higher than the C-index of the 8th AJCC N staging in the training set [0.641 vs. 0.629, *P* = 0.034] and testing set [0.671 vs. 0.658, *P* = 0.034]. LODDS staging may be a potential predictor of OS at 6 months [AUC = 0.687], 12 months (AUC = 0.692), and 48 months (AUC = 0.709), and LNR staging (AUC = 0.655) may be a potential predictor of OS at 24 months in AC patients. The predictive ability of LNR staging and LODDS staging were also found in different subgroups.

**Conclusion:**

The LNR and LODDS staging systems' predictive performance for OS of AC patients were superior to the 8th edition AJCC N staging system, especially in patients ages ≥65 or with higher tumor grade (grade II and III). The LNR staging and the LODDS staging were potential predictors for 24-month OS, and 6, 12, 24 and 48-month OS, respectively.

## Introduction

Ampullary carcinomas (ACs) account for 0.2% of all gastrointestinal tumors and approximately 20% of all periampullary carcinomas (1, 2). Although the overall incidence of ACs in Western countries is less than 0.5 cases per 100,000 people, there has been a significant increase in the incidence of ACs over the past few decades (3, 4). Radical surgery with lymphadenectomy is the main treatment for ACs, pancreaticoduodenectomy is the current standard, and approximately 50% of cases can be cured by surgery (5, 6). Given that the incidence of lymph node metastasis is between 20% and 50%, lymph node metastasis is an important factor affecting the survival of patients with ACs (7–10).

The 7th edition of the American Joint Committee on Cancer (AJCC) staging for ACs only determined the presence or absence of lymph node involvement, but the 8th edition of the AJCC classification and staging system refined the N staging (11). A study showed that the staging system based on positive lymph nodes ratio (LNR) [the ratio of positive lymph nodes to total lymph node number] has better predictive ability for the survival of ACs than the 8th edition of the AJCC staging system (12). Log odds of positive lymph node (LODDS) use pathological nodal data to stratify patients for differences in survival, and LODDS can help clinicians identify high-risk patients regardless of whether they are node-positive or not (8). The 8th edition AJCC N staging system, LNR and LODDS three lymph node staging systems are gradually applied in pancreatic and bile duct cancers (8, 13). However, there are no studies explore and compare the value of these lymph node staging systems in predicting the survival of ACs, and cannot provide a reference for lymph node staging in patients with ACs.

Therefore, in the present study, we aimed to analyze and compare the predictive performance of different lymph node staging systems (the 7th edition AJCC N staging system, the 8th edition AJCC N staging system, LNR and LODDS) for the survival of patients with ACs after surgery.

## Methods

### Study population

**Figure 1:**
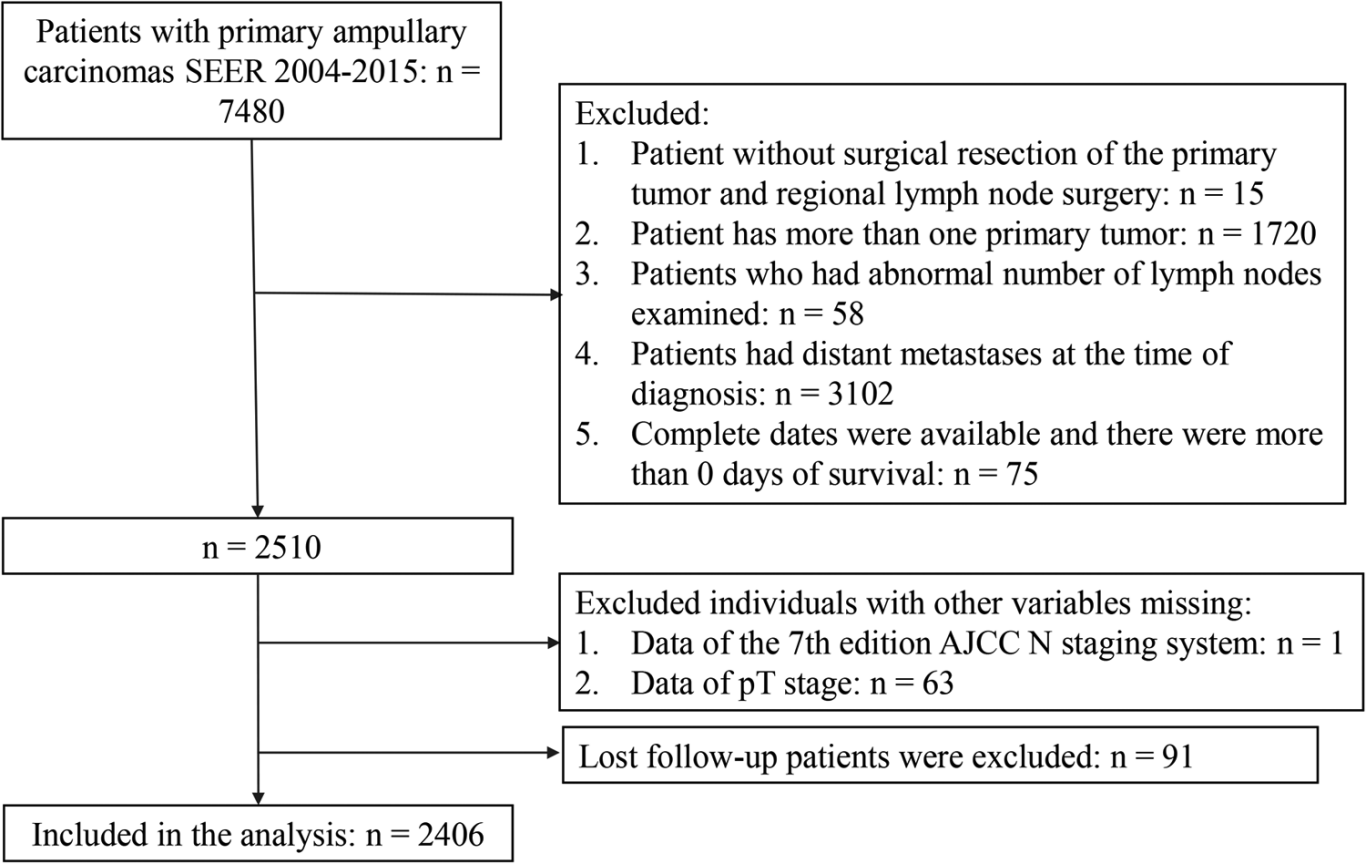
Flow chart of the study population.

This retrospective cohort study used data from the Surveillance, Epidemiology, and End Results (SEER) database from 2004 to 2015. The SEER collects cancer incidence data from the population-based cancer registry, which covers approximately 34.6% of the U.S. population, and is an open-source clinical database. Patients with primary ACs (C24.1-Ampulla of Vater) and ICD-O-3 Hist/behav = 3 were the study population. Patient records were extracted from SEER database for this study if they met the following criteria: (1) pathologically diagnosed as primary ACs; (2) with surgical resection of the primary tumor and regional lymph node surgery; (3) aged ≥18 years old; (4) only one primary tumor; (5) with records meeting AJCC criteria for the pathological staging of nodes. The excluded criteria were as follows: (1) patients who had abnormal number of lymph nodes examined; (2) patients had distant metastases at the time of diagnosis; (3) patients were diagnosed at autopsy only or through death certificates; (4) patients were not followed up actively; (5) complete dates were available and there were more than 0 days of survival; (6) patients had incomplete information for the variables included in the analysis were excluded. In total, 2,406 patients were enrolled in the final analyses. The flow chart was shown in Figure 1. The rate of loss to follow-up was 3.6%.

The SEER database is an open-access database, and all patient information has been de-identified, so this study was exempt from ethical review and patients' informed consent was waived.

### Outcome variable

The outcome was overall survival (OS), which was the time interval from diagnosis to the date of the most recent follow-up or date of death. The follow-up interval was monthly follow-up, the average follow-up time was 41.1 months, the minimum follow-up time was 2 months, and the maximum was 155 months.

### Four staging systems

The 7th edition AJCC N staging system stated N0 was no metastasis and N1 was positive lymph nodes ≥1. The 8th edition AJCC N staging for ACs is a three-category system, which included N0: no metastasis; N1: 1–3 positive lymph nodes; and N2: ≥4 metastatic lymph nodes. The pLN was defined as the number of positive lymph nodes (LN) and LNR was the ratio of pLN to total lymph node count (TLNC). X-tile was used to divide LNR, which was LNR = 0, 0–0.3, and >0.3 (14). The LODDS is defined as the natural logarithm of the probability ratio between LN with or without tumor invasion and is calculated as ln [(positive LN + 0.5)/(negative LN + 0.5)]. LODDS was divided into three categories using x-tile: LODDS < −2.4, −2.4 to −0.8, and >−0.8 (12).

### Data collection

The variables included age, race (Black, White, other, and unknown), sex (female and male), marital status (married, single/widowed/divorced and unknown), pT stage (T1/T2, T3 and T4), tumor size, grade [grade I (well-differentiated), grade II (moderately differentiated), grade III (poorly differentiated), grade IV (undifferentiated, anaplastic) and unknown], radiation (yes, no/unknown), chemotherapy (yes, no/unknown) and insurance (insured, uninsured and unknown).

### Statistical analysis

The study population was grouped by survival and death for descriptive analysis. Non-normal data were described by median and interquartile range [M (Q1, Q3)], comparison between groups was by Mann–Whitney *U* rank-sum test; enumeration data were described by number of cases and constituent ratio [*n* (%)], and the *χ*^2^ test was used to compare between groups. Univariate and multivariate Cox proportional hazard models were used to explore the association between four N staging systems (the 7th edition AJCC N staging system, the 8th edition AJCC N staging system, LNR and LODDS) and OS in AC patients. Crude model was the univariate Cox proportional hazard model and adjusted model was adjusted for variables including age, race, pT stage, tumor size, grade, radiation and insurance (*P* < 0.05). The study population were divided into a training set and a testing set in a ratio of 7–3. The C-index was calculated to compare the predictive performance of the four staging on OS in the training set and the testing set. The receiver operator characteristic curves (ROCs) of the predictive performance of OS at 6, 12, and 24 months for the training set and the test set were drawn. The analysis was performed in two subgroups based on the number of examined lymph nodes ≥12 and <12, which was to explore the predictive performance of the four staging systems in different subgroups.

R v. 4.0.3 (R Foundation for Statistical Computing, Vienna, Austria) and SAS v. 9.4 (SAS Institute, Cary, North Carolina) were used to conduct all statistical analyses.

## Results

### Characteristics of the study population

A total of 7,480 patients with primary ACs were extracted from the SEER 2004–2015. We excluded patient without surgical resection of the primary tumor and regional lymph node surgery (*n* = 15), patient has more than one primary tumor (*n* = 1,720), patients who had abnormal number of lymph nodes examined (*n* = 58), patients had distant metastases at the time of diagnosis (*n* = 3,102), complete dates were available and there were more than 0 days of survival (*n* = 75). There were 1 patient and 63 patients were excluded due to the missing data of the 7th edition AJCC N staging system and the pT stage, respectively. Lost follow-up patients were excluded (*n* = 91). A total of 2,406 patients were enrolled (Figure 1).

There were 1,178 survivals and 1,128 deaths. The demographic and clinicopathologic data were descripted in Table 1. In the eligible participants, the median age was 65.0 years old, 1,046 (43.5%) females and 1.360 (56.5%) males. The largest number of races were white [1,827 (75.9%)], followed by other races [379 (15.8%)] and Black [185 (7.69%)]. The number of 7th AJCC N0 stage and N1 stage was 1,103 (45.8%) and 1,303 (54.2%), respectively. The number of 8th AJCC N0 stage, N1 stage and N2 stage was 1,113 (46.3%), 874 (36.3%) and 419 (17.4%), respectively.

**Table 1:** The demographic and clinicopathologic characteristics of the AC patients in survival and death.

Characteristic	Total (*n* = 2,406)	Survival (*n* = 1,178)	Death (*n* = 1,228)	*P*
Age, years, M (Q1, Q3)	65.0 (56.0, 73.0)	63.0 (54.0, 71.0)	66.0 (58.0, 74.0)	<0.001
Race, *n* (%)				0.012
Black	185 (7.69%)	88 (7.47%)	97 (7.90%)	
White	1,827 (75.9%)	873 (74.1%)	954 (77.7%)	
Other	379 (15.8%)	205 (17.4%)	174 (14.2%)	
Unknown	15 (0.62%)	12 (1.02%)	3 (0.24%)	
Sex, *n* (%)				0.309
Female	1,046 (43.5%)	525 (44.6%)	521 (42.4%)	
Male	1,360 (56.5%)	653 (55.4%)	707 (57.6%)	
Marital status, *n* (%)				0.237
Married	1,503 (62.5%)	737 (62.6%)	766 (62.4%)	
Single/widowed/divorced	816 (33.9%)	391 (33.2%)	425 (34.6%)	
Unknown	87 (3.62%)	50 (4.24%)	37 (3.01%)	
Insurance, *n* (%)				<0.001
Insured	1,845 (76.7%)	998 (84.7%)	847 (69.0%)	
Uninsured	74 (3.08%)	47 (3.99%)	27 (2.20%)	
Unknown	487 (20.2%)	133 (11.3%)	354 (28.8%)	
Radiation, *n* (%)				<0.001
No/Unknown	1,829 (76.0%)	940 (79.8%)	889 (72.4%)	
Yes	577 (24.0%)	238 (20.2%)	339 (27.6%)	
7th AJCC N staging, *n* (%)				<0.001
N0	1,103 (45.8%)	688 (58.4%)	415 (33.8%)	
N1	1,303 (54.2%)	490 (41.6%)	813 (66.2%)	
8th AJCC N staging, *n* (%)				<0.001
N0	1,113 (46.3%)	692 (58.7%)	421 (34.3%)	
N1	874 (36.3%)	369 (31.3%)	505 (41.1%)	
N2	419 (17.4%)	117 (9.93%)	302 (24.6%)	
LNR staging, *n* (%)				<0.001
0	1,113 (46.3%)	692 (58.7%)	421 (34.3%)	
0–0.3	887 (36.9%)	393 (33.4%)	494 (40.2%)	
>0.3	406 (16.9%)	93 (7.89%)	313 (25.5%)	
LODDS staging, *n* (%)				<0.001
<−2.4	1,058 (44.0%)	691 (58.7%)	367 (29.9%)	
(−2.4, −0.8)	932 (38.7%)	393 (33.4%)	539 (43.9%)	
>−0.8	416 (17.3%)	94 (7.98%)	322 (26.2%)	
pT stage, *n* (%)				<0.001
T1/T2	288 (12.0%)	187 (15.9%)	101 (8.22%)	
T3	723 (30.0%)	445 (37.8%)	278 (22.6%)	
T4	1,395 (58.0%)	546 (46.3%)	849 (69.1%)	
Tumor size, mm, M (Q1, Q3)	22.0 (15.0, 35.0)	21.0 (15.0, 35.0)	24.0 (15.8, 35.0)	<0.001
Grade, *n* (%)				<0.001
Grade I (Well-differentiated)	110 (4.57%)	54 (4.58%)	56 (4.56%)	
Grade II (Moderately differentiated)	265 (11.0%)	153 (13.0%)	112 (9.12%)	
Grade III (Poorly differentiated)	1,247 (51.8%)	655 (55.6%)	592 (48.2%)	
Grade IV (Undifferentiated, anaplastic)	767 (31.9%)	309 (26.2%)	458 (37.3%)	
Unknown	17 (0.71%)	7 (0.59%)	10 (0.81%)	
Chemotherapy, *n* (%)				0.932
No/Unknown	1,247 (51.8%)	609 (51.7%)	638 (52.0%)	
Yes	1,159 (48.2%)	569 (48.3%)	590 (48.0%)	

LNR staging, lymph node ratio staging; AJCC, the american joint committee on cancer; LODDS staging, the log odds of positive lymph nodes staging.

Significant differences were found in age (*P* < 0.001), race (*P* = 0.012), insurance (*P* < 0.001), radiation (*P* < 0.001), pT stage (*P* < 0.001), tumor size (*P* < 0.001), and grade (*P* < 0.001) between survival group and death group (Table 1). Sensitivity analysis between the training set and testing set were summarized in Supplementary Table S1 and there was no significant difference.

### Association between four staging systems and OS of all participants

Table 2 revealed that the 7th edition AJCC N staging system, the 8th edition AJCC N staging system, LNR and LODDs were associated with OS (*P* < 0.001). In the 7th edition AJCC N staging system, N1 stage was associated with the OS compared with N0 stage [hazard ratio (HR) = 2.05, 95% confidence interval (CI): 1.80–2.33]. N1 stage [HR = 1.82, 95% CI: (1.58–3.24)] and N2 stage [HR = 2.75, 95% CI: (2.34–3.24)] of the 8th edition AJCC N staging system were related to OS compared with N0 stage in adjusted model. LNR staging (0–0.3) [HR = 1.72, 95% CI: (1.50–3.65)], LNR (>0.3) [HR = 3.11, 95% CI: (2.65–3.63)], LODDS (−2.4 to −0.8) [HR = 1.83, 95% CI: (1.60–3.81)] and LODDS (>−0.8) [HR = 3.25, 95% CI: (2.76–3.81)] were linked to OS in AC patients (Table 2).

**Table 2:** Association between four staging system and OS of all participants using univariate and multivariate Cox proportional hazard models.

Variables	Crude model		Adjusted model	
	HR (95% CI)	*P*	HR (95% CI)	*P*
**7th AJCC N staging**				
N0	Ref	Ref		
N1	2.46 (2.18–2.77)	<0.001	2.05 (1.80–2.33)	<0.001
**8th AJCC N staging**				
N0	Ref	Ref		
N1	2.10 (1.84–3.95)	<0.001	1.82 (1.58–3.24)	<0.001
N2	3.40 (2.92–3.95)	<0.001	2.75 (2.34–3.24)	<0.001
**LNR staging**				
0	Ref	Ref		
0–0.3	2.00 (1.75–2.28)	<0.001	1.72 (1.50–3.65)	<0.001
>0.3	3.84 (3.31–4.45)	<0.001	3.11 (2.65–3.65)	<0.001
**LODDS staging**				
<−2.4	Ref	Ref		
(−2.4, −0.8)	2.05 (1.79–2.34)	<0.001	1.83 (1.60–3.81)	<0.001
>−0.8	4.02 (3.46–4.68)	<0.001	3.25 (2.76–3.81)	<0.001

HR, hazard ratio; CI, confidence interval; Ref, reference; LNR staging, lymph node ratio staging; AJCC, the american joint committee on cancer; LODDS staging, the log odds of positive lymph nodes staging.

Model 1 was the univariate Cox regression model;.

Model 2 adjusted for age, race, pT stage, tumor size, grade, radiation, and insurance.

### The predicting ability of four staging systems for OS between training set and testing set

The predicting ability of four staging systems of OS in AC patients between training set and testing set was shown in Table 3. The C-index of the 7th AJCC N staging was significantly lower than the C-index of the 8th AJCC N staging in the training set [0.608, (95% CI: 0.591–0.625) vs. 0.629 (95% CI: 0.611–0.647), *P* < 0.001] and testing set [0.635, (95% CI: 0.611–0.659) vs. 0.658 (95% CI: 0.632–0.684), *P* < 0.001]. The C-index of the LODDS staging was significantly higher than the C-index of the 8th AJCC N staging in the training set [0.641, (95% CI: 0.623–0.659) vs. 0.629 (95% CI: 0.611–0.647), *P* = 0.034] and testing set [0.671, (95% CI: 0.644–0.698) vs. 0.658 (95% CI: 0.632–0.684), *P* = 0.034].

**Table 3:** The predicting ability of four staging systems for OS between training set and testing set.

Variables	C-index (95% CI) in the training set (*n* = 1,684)	C-index (95% CI) in the testing set (*n* = 722)	*P*
7th AJCC N staging	0.608 (0.591–0.625)	0.635 (0.611–0.659)	<0.001
8th AJCC N staging	0.629 (0.611–0.647)	0.658 (0.632–0.684)	Ref
LNR staging	0.637 (0.618–0.656)	0.668 (0.641–0.695)	0.103
LODDS staging	0.641 (0.623–0.659)	0.671 (0.644–0.698)	0.034

CI, confidence interval; Ref, reference; LNR staging, lymph node ratio staging; AJCC, the american joint committee on cancer; LODDS staging, the log odds of positive lymph nodes staging.

### The relationship between four staging systems and OS at 6, 12, 24 and 48 months between training set and testing set

**Figure 2:**
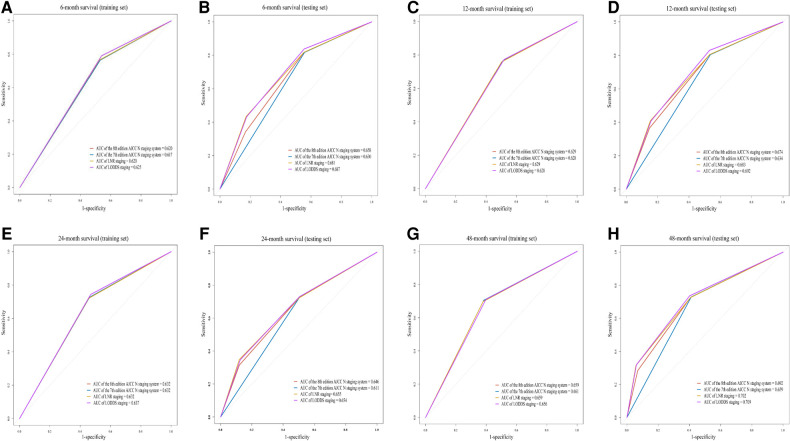
The receiver operator characteristic curves (ROCs) of four staging systems (the 7th edition AJCC N staging system, the 8th edition AJCC N staging system, LNR staging and LODDS staging) predicting OS at 6 months [training set (**A**) and testing set (**B**)], 12 months [training set (**C**) and testing set (**D**)], 24 months [training set (**E**) and testing set (**F**)] and 48 months [training set (**G**) and testing set (**H**)] in patients with ACs. AUC, area under the curve; LNR staging, lymph node ratio staging; AJCC, the american joint committee on cancer; LODDS staging, the log odds of positive lymph nodes staging.

Figure 2 shows the ROCs of four staging systems (the 7th edition AJCC N staging system, the 8th edition AJCC N staging system, LNR staging and LODDS staging) predicting OS at 6, 12, 24 and 48 months in training and testing sets. In the testing set, LODDS staging predicting OS was superior to other staging systems at 6 months [area under the curve (AUC) = 0.687], 12 months (AUC = 0.692), and 48 months (AUC = 0.709) in AC patients (Figure 2B,D and H). LNR staging (AUC = 0.655) and LODDS staging (AUC = 0.654) were both potential predictor of OS at 24 months in AC patients (Figure 2F).

### The association between four staging systems and OS of all participants in the subgroups of examined lymph nodes, age, gender and ACs grade

Subgroups analysis was shown in Table 4. The C-index of the 7th AJCC N staging was significantly lower than that of the 8th AJCC N staging in the group of examined lymph nodes ≥12 [0.620, (95% CI: 0.602–0.638)], age <65 years [0.629, (95% CI: 0.608–0.649)], age ≥65 years [0.600, (95% CI: 0.581–0.619)], male [0.603, (95% CI: 0.584–0.621)] and female [0.622, (95% CI: 0.600–0.644)]. The C-index of the LNR staging and LODDS staging were significantly higher than that of the 8th AJCC N staging in the group of age ≥65 years, male, female, grade II and grade III (all *P* < 0.05).

**Table 4:** The association between four staging systems and OS of all participants in the subgroups of examined lymph nodes, age, gender and ACs grade.

Variables	Groups	*n*	C-index (95% CI)	*P*
7th AJCC N staging	Examined lymph nodes ≥12	1,457	0.620 (0.602–0.638)	<0.001
8th AJCC N staging			0.656 (0.635–0.677)	Ref
LNR staging			0.651 (0.630–0.672)	0.175
LODDS staging			0.651 (0.630–0.672)	0.177
7th AJCC N staging	Examined lymph nodes <12	949	0.610 (0.589–0.631)	0.211
8th AJCC N staging			0.616 (0.594–0.638)	Ref
LNR staging			0.625 (0.603–0.647)	0.062
LODDS staging			0.623 (0.599–0.646)	0.121
7th AJCC N staging	Age <65	1,182	0.629 (0.608–0.649)	<.001
8th AJCC N staging			0.651 (0.629–0.674)	Ref
LNR staging			0.659 (0.637–0.682)	0.071
LODDS staging			0.656 (0.633–0.679)	0.260
7th AJCC N staging	Age ≥65	1,224	0.600 (0.581–0.619)	<.001
8th AJCC N staging			0.617 (0.596–0.637)	Ref
LNR staging			0.637 (0.615–0.658)	<.001
LODDS staging			0.648 (0.626–0.670)	<.001
7th AJCC N staging	Male	1,360	0.603 (0.584–0.621)	<.001
8th AJCC N staging			0.625 (0.605–0.645)	Ref
LNR staging			0.651 (0.630–0.673)	<.001
LODDS staging			0.664 (0.642–0.685)	<.001
7th AJCC N staging	Female	1,046	0.622 (0.600–0.644)	0.003
8th AJCC N staging			0.636 (0.613–0.660)	Ref
LNR staging			0.649 (0.625–0.673)	0.003
LODDS staging			0.659 (0.634–0.685)	<.001
7th AJCC N staging	Grade I	265	0.605 (0.556–0.653)	0.942
8th AJCC N staging			0.606 (0.558–0.654)	Ref
LNR staging			0.613 (0.562–0.664)	0.613
LODDS staging			0.623 (0.567–0.679)	0.232
7th AJCC N staging	Grade II	1,247	0.617 (0.597–0.638)	<.001
8th AJCC N staging			0.633 (0.611–0.655)	Ref
LNR staging			0.647 (0.624–0.670)	0.001
LODDS staging			0.652 (0.628–0.675)	<.001
7th AJCC N staging	Grade III	767	0.581 (0.559–0.603)	<.001
8th AJCC N staging			0.609 (0.583–0.635)	Ref
LNR staging			0.642 (0.616–0.669)	<.001
LODDS staging			0.662 (0.635–0.688)	<.001

CI, confidence interval; Ref, reference; LNR staging, lymph node ratio staging; AJCC, the american joint committee on cancer; LODDS staging, the log odds of positive lymph nodes staging.

## Discussion

In this study, we found that the N1 stage and N2 stage of the 8th edition AJCC N staging system, LNR staging (0–0.3), LNR (>0.3), LODDS (−2.4 to −0.8) and LODDS (>−0.8) were associated with OS in AC patients. The C-index of the 7th AJCC N staging was the lowest in the four staging systems. The C-index of the LODDS staging was significantly higher than the C-index of the 8th AJCC N staging. LODDS staging's prediction of OS at 6 months, 12 months, and 48 months was superior to the other three staging systems, and LNR staging was a potential predictor of OS at 24 months. In addition, the predicting ability of LNR staging and LODDS staging was superior to the 7th AJCC N staging in AC patients who aged ≥65 years or with higher tumor grade (grade II and III).

In the current the 8th edition AJCC staging system, N stages are divided into N0 (0 positive lymph nodes), N1 (1–3 positive lymph nodes), and N2 (4 or more positive lymph nodes) (11). Modified on the basis that the 7th edition AJCC N staging system only determined the presence or absence of positive lymph nodes. Results in our study showed that the C-index in the 7th edition AJCC N staging system was lower than the C-index in the 8th edition AJCC N staging system. LNR staging has been proven to be a prognostic factor in patients with AC, which was better than the 8th edition AJCC N staging system (15). Kim et al. (16) retrospectively analyzed 71 patients with AC who underwent adjuvant chemotherapy after radical resection and found that LNR > 0.15 was an independent risk factor for OS. In an analysis of 212 AC patients in Taiwan who underwent radical surgery, LNR > 0.056 suggested poor DFS and OS (17). The 8th edition AJCC N staging depends only on the number of positive lymph nodes and does not consider the number of lymph nodes (or negative nodes) examined. When the number of positive lymph nodes is the same, patients with insufficient number of examined lymph nodes may have a poorer prognosis, a phenomenon of insufficient staging called stage migration (18). LODDS staging considers the number of positive and negative lymph nodes, so it can also reduce the possibility of stage migration due to insufficient number of examined lymph nodes. When all lymph nodes examined were positive, the LNR value did not increase with the number of positive lymph nodes. When all lymph nodes examined were negative, LODDS values decreased with increasing number of negative lymph nodes (13). LODDS has unique value for risk stratification of node-negative patients, which is not available with LNR and 8th AJCC N staging systems (19). Results in our study stated that the C-index of the LODDS staging was significantly higher than the C-index of the 8th AJCC N staging and the LODDS staging predicting ability of OS at 6 months, 12 months, and 48 months was superior to the other three staging systems. Huang et al. (8) showed that the LODDS staging system seems to have superior predictive survival compared with the AJCC staging system and the LNR staging. However, the predictive performance of the LODDS remains controversial. Morales–Oyavid et al. (19) showed that the prognostic value of the LNR and LODDS classifications was not significantly improved in pancreatic ductal adenocarcinoma compared with the 8th edition AJCC staging system. Gao et al. (13) concluded that the 8th edition AJCC N staging was more reliable than LNR and LODDS staging in predicting cause-specific survival in pancreatic neuroendocrine tumors.

There were many factors affecting the survival of patients with ACs, such as age (20), differentiation (17), tumor size (21), etc., thereinto, lymph node status was an important factor affecting the survival of ACs patients (21–23). As the number of positive lymph nodes increases, patients with ACs have a poor prognosis (21, 24). It is also recommended that at least 12 lymph nodes be removed in patients with AC according to the recommendations of the AJCC 8th edition (9, 17). In current study, we found no significant difference among four staging systems of predicting OS in ACs patients. However, the LNR staging and LODDS staging may be potential predictors of OS compared with the 7th edition AJCC N staging system especially in AC patients aged ≥65 years or with higher tumor grade (grade II and III). In Barauskas et al. (25) study, AC patient age at the cutoff of 70 years emerged as a potential risk factor affecting survival. Bathe et al. (26) retrospectively analyzed a group of patients with various periampullary tumors who were aged more than 65 years at the time of surgery, and the 5-year survival appeared not much different from that in younger populations. Lewis et al. (27) suggested that higher lymph node counts in younger patients are due to increased lymph node hyperplasia (LNH) leading to improved pathologic identification which aiding in identification of LN within surgical specimens by pathologists. This could be a direct reflection of the age-related changes that exist in the immune system resulting in more robust response in younger patients. Further exploration is needed on the prediction of OS by LNR and LODDS in AC patients of different ages. In addition, we also found that LNR and LODDS staging better predicting OS in higher grade of AC patients. Grading information is important for predicting prognosis in pancreatic neuroendocrine tumors patients, and however, whether factors combination have greater power should be further explored (28). Li et al. (29) also indicated that in addition to LODDS stage, tumor grade, SEER historic stage, and tumor size were also independent risk factors for distal cholangiocarcinoma patients, and the prognostic value of these factors has been verified in a variety of tumors (30–32). Since the predicting ability of LNR and LODDS staging based on the positive lymph nodes ratio may be superior to other staging, but the association between AC grade and LNR and LODDS for predicting OS in AC patients still needed discussion.

The strengths of this study were as follows. To the best of our knowledge, this was the first study to explore and compare the predictive performance of the four staging systems in ACs patients after surgery. And the subgroup analysis was performed with a cut-off value of 12 for the number of lymph nodes examined to explore the survival prediction of different lymph node stages under the number of lymph nodes examined. But, there were a few limitations in our study. First, the current four lymph nodes staging systems we studied is based on the number of lymph nodes examined, so the current lymph node staging was used in patients with AC who have undergone surgery. Second, although we adjusted Although we adjusted for some demographic and clinicopathological variables, other variables were not available due to database limitations, such as other treatments or nursing care during follow-up, which may have affected OS. Third, since our data were derived from the SEER database and the data were retrospective, they were exposed to selection bias. Prospective studies with large samples are needed for exploration and comparison, and external validation of our results is required.

## Conclusion

For patients with AC, the LNR and LODDS staging systems appear to have better predictive performance for OS compared with the 8th edition AJCC N staging system partly, especially in patients ages ≥65 or with higher tumor grade (grade II and III). In particular, the LNR staging for 24-month OS prediction, and the LODDS staging for 6, 12, 24 and 48-month OS prediction showed slightly better predictive performance.

## Data Availability

Publicly available datasets were analyzed in this study. This data can be found here: SEER database, https://seer.cancer.gov/.
